# Marburg virus pathogenesis – differences and similarities in humans and animal models

**DOI:** 10.1186/s12985-019-1272-z

**Published:** 2019-12-30

**Authors:** Kyle Shifflett, Andrea Marzi

**Affiliations:** Laboratory of Virology, Division of Intramural Research, National Institute of Allergy and Infectious Diseases, Rocky Mountain Laboratories, National Institutes of Health, 903 South 4th Street, Hamilton, MT 59840 USA

**Keywords:** MARV, Filovirus, Human pathogenesis, Animal models

## Abstract

Marburg virus (MARV) is a highly pathogenic virus associated with severe disease and mortality rates as high as 90%. Outbreaks of MARV are sporadic, deadly, and often characterized by a lack of resources and facilities to diagnose and treat patients. There are currently no approved vaccines or treatments, and the chaotic and infrequent nature of outbreaks, among other factors, makes testing new countermeasures during outbreaks ethically and logistically challenging. Without field efficacy studies, researchers must rely on animal models of MARV infection to assess the efficacy of vaccines and treatments, with the limitations being the accuracy of the animal model in recapitulating human pathogenesis. This review will compare various animal models to the available descriptions of human pathogenesis and aims to evaluate their effectiveness in modeling important aspects of Marburg virus disease.

## Background

Marburg virus (MARV) is the causative agent of Marburg virus disease (MVD) in humans, with a case fatality rate ranging from 23 to 90%, depending on the outbreak [1]. MARV is a member of the *Filoviridae* family, which consists of the genera *Marburgvirus, Ebolavirus, Cuevavirus, Striavirus, and Thamnovirus* [2, 3]. The family*,* known as filoviruses, contains several viruses that are known to cause hemorrhagic, often lethal disease in humans and nonhuman primates (NHPs) all of which are within the *Marburgvirus* or *Ebolavirus* genera. *Cuevavirus, Striavirus,* and *Thamnovirus* are not known to cause disease in humans or NHPs. Filoviruses have a non-segmented RNA genome in the negative sense, encoding for seven open reading frames; nucleoprotein NP, virion protein (VP) 35, VP40, glycoprotein GP, VP40, VP24, and viral polymerase L [4]. The filovirus genome is packaged into a unique filamentous virion, approximately 790 to 970 nm in length and 80 nm in width [5].

Within the genus *Marburgvirus* there is one species, *Marburg marburgvirus,* which is represented by two viruses; MARV and Ravn virus (RAVV) [6]. Although generally less well known than its cousin Ebola virus (EBOV), MARV was the first filovirus discovered following outbreaks in Germany and Yugoslavia (now Serbia) in 1967 [7]. Following its discovery, MARV cases were sporadically identified in Africa. However, in 1999 an outbreak was identified in the Democratic Republic of Congo, where multiple spillover events into the human population are thought to have taken place over the course of 2 years. This outbreak resulted in a total of 154 cases, with a case fatality rate of 83% [8]. In 2005, the largest documented outbreak of MARV occurred in Angola with 252 documented human infections and 227 deaths; a case fatality rate of 90% [9]. Outbreaks have continued to pop up since 2005, with a 2007 outbreak in Uganda, two instances in 2008 that involved tourists visiting Uganda returning home to the United States and Netherlands with MVD, and outbreaks in Uganda in 2012, 2014, and 2017 [1]. MARV was quickly recognized as a pathogen of extreme global importance and is currently classified as a Risk Group 4 pathogen by the World Health Organization and as a Select Agent by the US Centers for Disease Control and Prevention. There are no licensed vaccines or treatments for MVD, partly due to the difficulty of performing clinical trials given the severity, infrequency, and rural nature of MVD outbreaks. Animal models of MVD are necessary to develop and test potential vaccines and treatments, and the ability of these models to reflect human pathogenesis is essential to moving forward into clinical trials.

## Main text

### MARV reservoir

All recorded MARV outbreaks have originated in Africa, excluding laboratory infections, where the virus is thought to be maintained in a natural reservoir [10]. Several bat species have been implicated in being a reservoir host for filoviruses [11], and there is strong evidence that *Rousettus aegyptiacus*, the Egyptian fruit bat, serves as a reservoir for MARV. Several cases of tourists and miners most likely acquiring MARV in caves populated by *R. aegyptiacus* have been reported [12–14]. Live virus was isolated from *R. aegyptiacus* bats within the Kitaka Cave, Uganda, the place where miners that had been diagnosed with MVD had worked [15].

Experimental infection of *R. aegyptiacus* bats with MARV yielded no outward symptoms of infection but was associated with a mild immune response and detection of viremia in multiple organs, with viral shedding detected in oral and rectal swabs [16–18]. Despite the shedding of virus and maintenance of viremia, there was a lack of transmission to susceptible *R. aegyptiacus* bats when cohoused with infected bats for up to 42 days [17]. In addition, the livers of MARV-infected bats showed hepatocellular necrosis and changes in inflammatory cells beginning at 3 days post infection (dpi) and progressed through to 7 dpi [17]. Hepatocytes and macrophages of the liver contained MARV antigen, as did macrophages of the spleen. This is reflected by increased alanine aminotransferase levels measured in infected bats, indicating liver damage [17]. Subcutaneous macrophages and other cells of the subcutaneous tissue from the site of inoculation also contained MARV antigen, as did small numbers of cells in the draining lymph nodes [19]. These pieces of information collectively support the narrative of *R. aegyptiacus* being a reservoir host of MARV and provide evidence for possible routes of transmission.

### MARV human pathogenesis

**Fig. 1:**
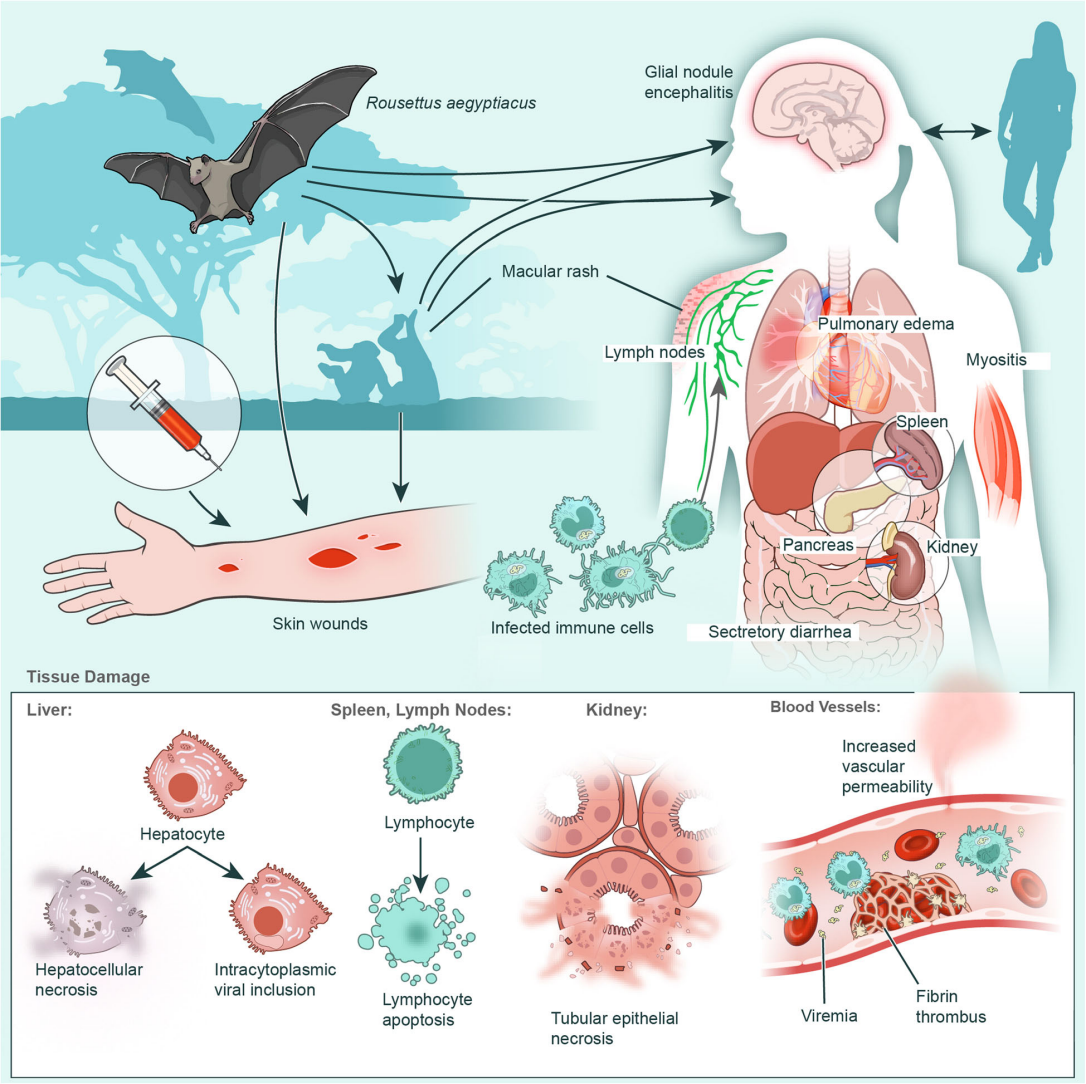
MARV pathogenesis in humans. Transmission and virus spread in the human body are depicted

There are few detailed clinical descriptions of MVD, due to the rural and severe nature of most outbreaks in Africa, and the availability of pathological and laboratory data from patients is limited. The detailed descriptions that do exist come from the initial outbreak in Marburg, Germany [20–22], an outbreak in Johannesburg, South Africa, involving three patients [23], and a few smaller, isolated cases and outbreaks originating elsewhere in Africa [13, 24–28]. In the following descriptions of various cases and outbreaks have been compiled to create an overall description of MARV pathogenesis in humans (Fig. 1).

Similar to many other infectious diseases, cases of MVD begin with flu-like symptoms such as chills, fever, headache, sore throat, myalgia, joint pain, and malaise, 2–21 days after the initial infection. Within 2–5 days of the first symptoms, patients can experience abdominal pain, nausea, vomiting, watery diarrhea, and lethargy. On days 5–7, the intensity of the disease increases, and may include a maculopapular rash spreading from the torso to the limbs, conjunctivitis, sustained fever, and symptoms of hemorrhagic fever, such as mucosal bleeding, petechiae, blood in the stool and vomitus, and bleeding from venipuncture sites. The maculopapular rash begins as small, dark red spots around hair follicles of the trunk and sometimes upper arms, developing into a diffuse rash, and can become a dark erythema that covers the face, neck, chest, and arms. Neurological symptoms such as confusion, agitation, increased sensitivity, seizures, and coma can occur in later stages of the disease, and all patients of the initial outbreak in Marburg, Germany, were described as having a sullen, negative, and slightly aggressive behavior [29]. Increases in alanine and aspartate aminotransferase (ALT and AST) and increased serum creatinine levels indicate hepatic and kidney damage [20, 23, 24]. Disseminated intravascular coagulation (DIC) [22, 28], lymphopenia, and thrombocytopenia [20] appear typically within 1 week of the first symptoms. In the late stages of disease, lymphopenia is offset by neutrophilia [30]. Patients either recover from their illness or die of dehydration, internal hemorrhage, organ failure, or some combination of systemic factors aided by a dysregulated immune response to the virus. Patients that survive typically don’t experience the severe late stage symptoms, but may experience sequelae such as arthritis, conjunctivitis, myalgia, and symptoms of psychosis during and after recovery [20]. Experiments with cultured cells from survivors indicate a proper adaptive response mounted by immune cells to the virus infection. In addition, serum samples from survivors showed IgG responses to MARV NP and GP, with two of the patients having significant neutralizing antibody titers. The neutralizing antibody titer diminished over time, with the decrease beginning at 21 months post infection (mpi) and dropping below detectable limits at 27 mpi [31].

Autopsies of RAVV-infected patients with lethal outcomes showed swelling of the heart, brain, spleen, kidneys, and lymph nodes, as well as hemorrhage of mucous membranes, soft tissues, and various other organs. All tissues examined had some form of hemorrhage, and focal necrosis was found on almost all organs and was especially prominent in hepatic and lymphatic tissues, as well as the testis and ovaries [32]. Damage to the liver tissue was severe, and there was extensive hepatocellular swelling and degeneration. Basophilic cytoplasmic inclusions were found in eosinophils near areas with necrosis and were positive for viral antigen [32]. Additionally, there were hepatocytes and Kupffer cells that had inclusions similar to the ones found in eosinophils, though most Kupffer cells were unidentifiable in the tissues analyzed. In the spleen, there was moderate necrosis in both the red and white pulp, with lymphoid depletion evident in the white pulp. The red pulp had deposits of fibrin and cellular debris. The sinuses had cellular debris and granular material deposited, along with a small amount of fibrin [32]. Hemorrhage and severe necrosis were observed in the germinal centers. Viral antigen was present in the marginal zone of the red pulp and in macrophages, but was not present in the germinal centers, despite the severe necrosis. In the lymphatic organs and mucous membranes of the stomach and intestines, there was a high number of plasma cells and monocytes. There was a marked depletion of lymphocytes, now thought to be the product of bystander apoptosis rather than direct infection [32]. The kidneys were swollen, pale, and hemorrhagic, and there was tubule necrosis and parenchymal damage. Macrophages in the intestines and kidney contained what looked like viral inclusions. The alveoli of the lungs were congested, hemorrhaged, and contained alveolar macrophages surrounded by fibrin, and occasionally stained positive for viral antigen. Three of the five autopsied cases in the outbreak in Marburg, Germany had glial nodule encephalitis, spread throughout the brain [23, 29, 33]. Taken together, the disease manifestations in the organs fit with the course of disease and give insight into the potential of sequelae experienced by MVD survivors.

### MARV NHP model

There are four main species of NHP that have been used in MARV research; the cynomolgus macaque (*Macaca fascicularis*), rhesus macaque (*Macaca mulatta*), common marmoset *(Callithrix jacchus)*, and the African green monkey (*Cercopithecus aethiops*). Imported African green monkeys harboring MARV were the cause of the 1967 outbreak in Germany and Yugoslavia (now Serbia), leading to many initial studies of MVD in NHPs being conducted in African green monkeys. The literature involving infections of African green monkeys with MARV lacked detailed histological data, and the details of their inoculation route and strain were often not specified [22]. For this reason, they have not been included in this review.

**Fig. 2:**
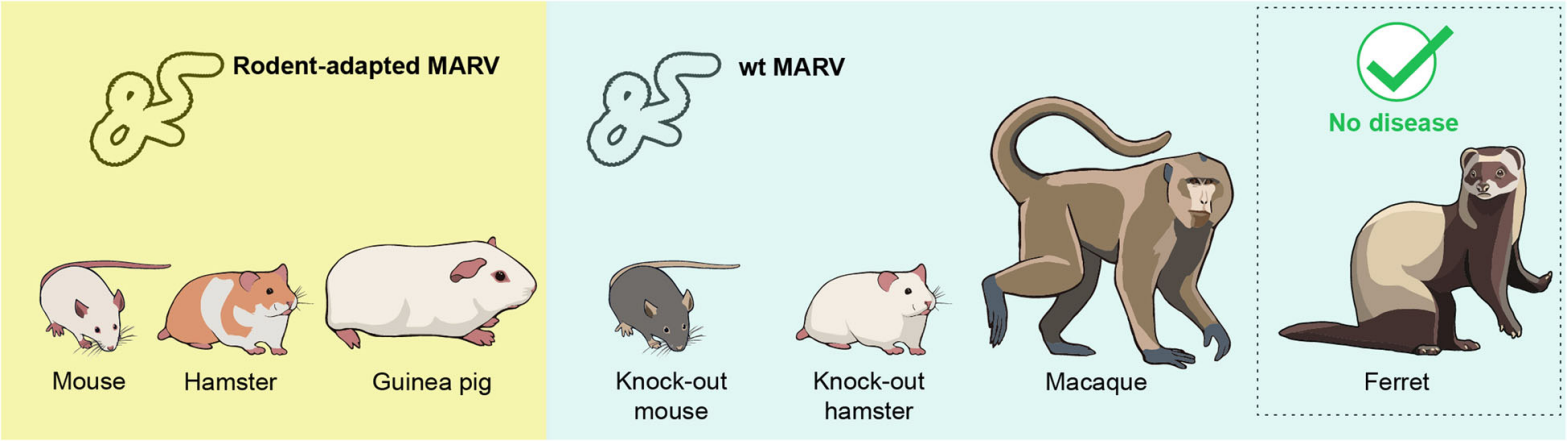
Commonly used animal models for MARV research. Infection with rodent-adapted viruses (left) and wild-type (wt) MARV (right) lead to disease in all animals tested with the exception of the ferret

Cynomolgus and rhesus macaques are the most common NHP models of MVD used in current research (Fig. 2) as they develop very similar disease and pathology compared to human MVD (Tables 1 and 2). The overall pathology for MVD in these two NHPs is very similar, with a few noteworthy differences. Compared to rhesus macaques, cynomolgus macaques infected with MARV experience a more accelerated disease course [34]. Different strains of MARV seem to produce a similar disease course, with the exception of MARV-Angola, which is associated with a more rapid onset and time to euthanasia in several NHP studies [34–36]. Doses used to inoculate NHPs with MARV can vary from experiment to experiment; however, it has been shown that as the inoculation dose increases, the length of the disease course decreases and always results in a lethal outcome [37–39]. Several inoculation routes have been used to challenge NHPs with MARV, and studies have found that the overall outcome is almost the same for all routes [22]. For example, in NHPs inoculated via the aerosol route, but not the intramuscular (i.m.) route, an early infection of the lymphoid tissue of the lung occurs, but both routes result in a systemic infection with similar levels of dissemination [40]. Fever generally appears 4–6 dpi, followed by anorexia, weight loss, and the development of a maculopapular rash of varying severity. Hemorrhagic symptoms began 6–7 dpi and consisted of bleeding from the gums and venipuncture sites. At 1–2 days before the humane endpoint, which generally occurred at 7–12 dpi, animals displayed lethargy, lack of reaction or interest in environment, diarrhea, a drop in body temperature, and dehydration [41–47]. Viral loads in the blood, liver, and spleen were the highest, but virus was detected in most tissues sampled, including the brain, indicating a systemic infection [44]. In the blood, there was a reduction in lymphocytes until 6–7 dpi [37, 42, 45, 48], when overall leukocyte counts spiked, due to lymphocytosis and neutrophilia [44]. Thrombocytopenia was observed in the early to middle phase of disease, sometimes recovering in the middle to late stage of disease [37, 42]. Blood analysis showed increases in AST, ALP [35, 41–43], and total bilirubin [35, 43, 45], indicating hepatic damage. Increases in blood urea nitrogen (BUN) [42, 43] and creatine indicated kidney damage [41]. Decreases in protein-C activity [41] and increases in D-dimers and blood coagulation times (PT and aPTT) indicated DIC [44]. Serum samples were analyzed for cytokine responses to MARV infection, which began 6–8 dpi and continued until the humane endpoint. Initially, there were increased levels of IFN-α, IL-6, MIP-1α, MIP-1β, MCP-1, and eotaxin. Later in the course of disease, IFN-β, IFN-γ, IL-1R, IL-2R, IL-8, IL-6, IL-12 p40/p70, IL-13, IL-1β, and TNF-α were detected at increased levels [36, 44]. This influx of pro- and anti-inflammatory cytokines indicates that MARV causes a dysregulation of the host immune system, similar to that of septic shock in bacterial infections. A similar dysregulated immune response contributing to pathology and severity of disease has been described for EBOV [49].

**Table 1:**
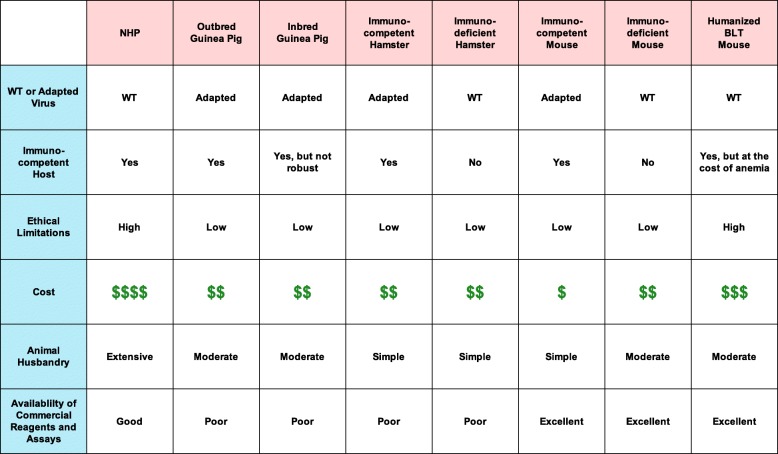
Comparison of various characteristics between animal models of MVD. $$ represents a higher cost than $, and lower cost than $$$. $$$$ most expensive. WT wild-type

**Table 2:**
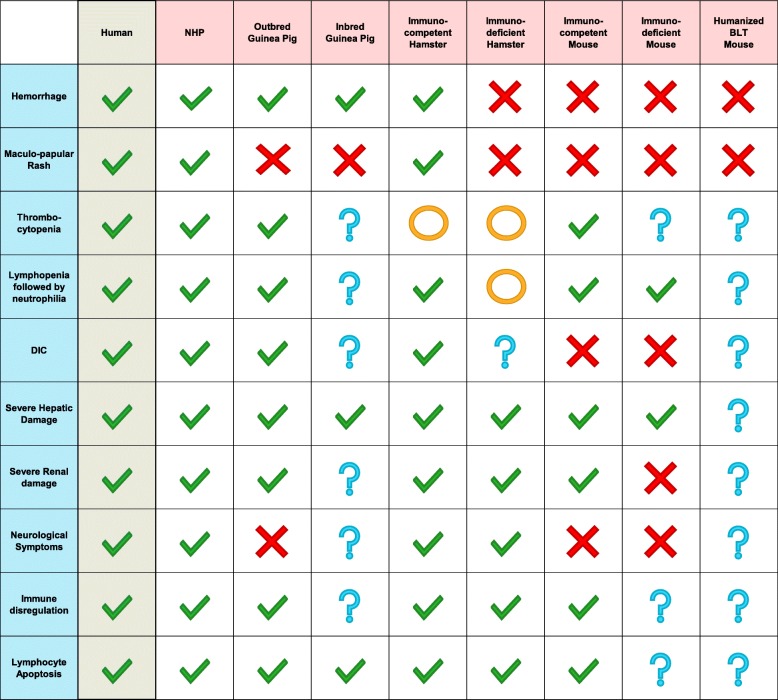
Animal models of MVD and the pathologies they present during the course of disease, as compared to humans. “X” means the pathology is not present; the open circle indicates not significant presence of the feature; a check mark means the pathology is present, and a question mark (?) represents a gap in knowledge for this pathological feature in this specific animal model

Studies that reported detailed necropsy, histology, and immunohistochemistry [35, 37, 44] found enlarged, congested, and discolored livers that contained increased numbers of mononuclear cells, Kupffer cells containing debris and MARV antigen, eosinophils with viral inclusion bodies, MARV antigen in the sinusoidal lining, and lesions of necrotic hepatocytes. The spleen had MARV antigen throughout the red pulp, with mononuclear cells and red pulp macrophages containing MARV antigen, and apoptotic lymphocytes, with a noticeable increase in dendritic cells positive for MARV antigen starting at 4 dpi. As the disease progressed, necrotic lesions grew in size and number, there was cellular necrosis and red blood cells (RBCs) in the red pulp, lymphopenia in the white pulp, and fibrin deposition in the red pulp and marginal zones. The lymphocytes of the spleen were negative for MARV antigen, despite the obvious degeneration of lymphocytes, supporting the bystander apoptosis seen in human MVD [32]. The lymph nodes had evidence of hemorrhage, lymphoid depletion, and contained tingible body macrophages [44]. MARV antigen was detected in medullary, subcapsular, and cortical sinuses, as well as in areas of lymphocytolysis. The few dendritic cells remaining no longer took on a dentritiform appearance. Fibrin deposits were detected in the vessels of the kidney. Within the lungs there was hemorrhage, edema, and fibrin in the alveoli, indicating interstitial pneumonia [35, 37, 46]. Immunohistochemical analysis of tissues showed that the first cells to show MARV antigen were Kupffer cells of the liver and dendritic cells and macrophages of the spleen [44].

Marmosets (*Callithrix jacchus*) are NHPs that weigh less than 500 g and are frequently used in viral disease modeling. Their small size is particularly suited to high level containment studies, where limited space is always a factor. However, the model has limitations in regard to sampling as only small blood volumes can be drawn at a time. Marmosets infected with MARV-Popp and Marv-Musoke succumbed to infection within 8–10 dpi. Disease progression, gross pathology, blood analysis, and histopathology were all in line with what is observed in humans and the previously described NHPs. The sole exception is the inconsistency of maculopapular rash, with one study having no observable rashes, and the other study having rashes in 2 of 8 animals. The marmoset represents a small NHP alternative to established NHP models of MVD, and accurately portrays the pathogenesis observed in human MVD [50, 51]. While a marmoset is less expensive compared to a macaque, its small size limits sample quantity; in addition, marmosets can develop diseases like hepatitis leading to premature euthanasia due to study-unrelated complications and are therefore not as commonly used as macaques.

### MARV mouse model

Mice are and have always been a staple of in vivo research, and the ease of acquiring and handling mice is appealing, particularly in high containment laboratories. Similar to other filoviruses, infection with MARV is not lethal for immunocompetent adult mice but does cause lethal disease in suckling mice, severe combined immunodeficient (SCID) mice, and mice lacking a type 1 interferon response (IFNAR^−/−^ or STAT1^−/−^) [52] (Fig. 2). STAT1^−/−^ mice infected with MARV-Musoke succumbed within 7 dpi, but when immunized with a filovirus vaccine produced antibody responses comparable to immunocompetent mice. The STAT1^−/−^ mice developed lymphopenia and there was MARV antigen in macrophages. Necrosis of hepatocytes was noted starting 3 dpi [53]. RAVV and MARV isolates Ci67, Musoke, and Angola were serially passaged through SCID mice 10 times, resulting in a significantly reduced time of death for every isolate [54]. Further passaging of the SCID-adapted RAVV in immunocompetent BALB/c mice resulted in a mouse-adapted RAVV (ma-RAVV) that was able to infect and cause lethal disease in adult immunocompetent BALB/c mice after 14 passages, a total of 24 passages from wild-type (wt) [55]. BALB/c mice were then inoculated with 1000 or 100,000 PFU of ma-RAVV. A variety of infection routes were tried, including subcutaneous, intranasal, intramuscular (i.m.), footpad, and intraperitoneal (i.p.), but only the i.p. route resulted in lethal disease. BALB/C mice infected with ma-RAVV became lethargic and hunched, with no evidence of hemorrhagic symptoms or maculopapular rash, and all mice succumbed to infection within 8 days.

A similar approach was taken using MARV-Angola, a strain isolated from the 2005 MARV outbreak in Angola. MARV-Angola was serially passaged through SCID mice 24 times using liver homogenates [56]. This resulted in a mouse-adapted MARV-Angola (ma-MARV-Ang) that caused uniform lethality in SCID mice within 8 dpi, via the i.p. route. Similar results were achieved when BALB/c mice were challenged with ma-MARV-Ang, but 100% lethality was only achieved through the i.p. route. None of the mice showed evidence of maculopapular rash, other hemorrhagic symptoms, or DIC; all common symptoms of MVD in humans and NHPs (Tables 1 and 2). Blood analysis of BALB/c mice infected with ma-MARV-Ang and ma-RAVV showed early lymphopenia and thrombocytopenia, with late neutrophilia, elevated ALT and ALP, amylase, BUN, and total bilirubin levels [56] (Tables 1 and 2). Liver, spleen, and blood samples had the highest viral titers, with varying levels detected in the kidney, lung, intestines, and brain by 3 dpi. Both pro- and anti-inflammatory cytokines were detected in the plasma at varying levels and different times, increasing as disease progressed, indicating a dysregulation of the immune system [56]. Infection with ma-MARV-Ang and ma-RAVV seemed to cause systemic infection of BALB/c mice, leading to multiorgan failure, an outcome similar to MVD in humans. This is supported by the necropsy of the BALB/c mice, which showed enlarged, discolored livers with extensive hepatocellular necrosis and inclusion bodies within eosinophils [55, 56]. The spleens were enlarged and had extensive necrosis and lymphocyte depletion. The kidneys were discolored, and intestinal hyperemia was observed. Ma-MARV-Ang had a total of 11 amino acid changes when compared to wt MARV-Angola, with 6 mutations in VP40, 2 mutations in VP35, 1 mutation each in GP, VP30, and VP24 [57]. It is not known which of these changes allows ma-MARV-Ang to cause lethal disease in BALB/c mice, but it has been shown that MARV VP40 is responsible for the INF antagonism in MARV infection of human cells by inhibition of the Jak1 pathway [58]. Specific amino acid changes to VP40 necessary for RAVV and MARV-Ci67 to inhibit IFN signaling in mouse cells have been identified as being strain- and species-specific [58, 59].

Mice provide a relatively accurate and convenient model for mammalian diseases, but there are considerable differences between the human and mouse immune systems that can present challenges, especially when testing vaccines and therapeutics against agents that interact with immune cells and pathways, as MARV does [60]. The immunocompetent and immunodeficient mouse models discussed above each have drawbacks, due to the resistance of mice to wt filovirus infection. Immunocompetent mouse models for MARV require an adapted strain of virus that differs from the human/NHP virus, and immunodeficient mouse models lack a robust immune response to the pathogen and to vaccination. Vaccines against EBOV and MARV using live-attenuated vesicular stomatitis virus (VSV) as a backbone were tested in STAT1^−/−^, only to find that some of the recombinant VSV (rVSV) vaccines and rVSV wt caused lethal disease due to the lack of a functional IFN response able to control VSV replication [61]. To address these shortcomings, an immunodeficient mouse strain with *Rag2, γ*_*c*_*,* and *CD47* genes knocked out was humanized using a bone marrow, liver, thymus (BLT) method. This mouse produces human dendritic cells, monocytes, monocyte-derived macrophages, natural killer cells, B cells, and T cells. These triple knockout BLT (TKO-BLT) mice were i.m. inoculated with MARV-Angola. TKO-BLT mice lost weight starting 16 dpi, and disease resulted in morbidity, but not uniform lethality. Two mice infected are suspected to have died from anemia related to the BLT method. Viral antigen was detected in the liver, infecting mostly murine cells. Activation of dendritic cells, T cells, monocyte derived macrophages, and monocytes was observed [62]. This model represents a unique opportunity to study filovirus interactions with human immune cells in vivo*.* However, due to the extensive cost and ethical concerns of generating these mice they are not an adequate model for countermeasure evaluation.

### MARV Guinea pig model

One of the first in vivo experiments with MARV was conducted by inoculating Hartley guinea pigs with whole blood from a patient from the 1967 outbreak in Marburg, Germany [46]. After an incubation period of 4–10 days, the guinea pigs developed a febrile illness, stopped eating and drinking, lost weight, and become lethargic; however, most recovered. After 8 passages through guinea pigs using whole blood, 100% of guinea pigs inoculated died 7–9 dpi [46]. The guinea pigs exhibited the same symptoms as in the first infection, but blood taken before death occasionally failed to coagulate, mirroring the clotting abnormalities in human MVD. Necropsies revealed enlarged spleens with severe damage to lymphoid tissue, RBCs and degenerating leucocytes in the red pulp, and macrophages and coagulated material in the sinuses. The liver was soft and discolored, with focal necrotic lesions, and there were basophilic granules in the cytoplasm of cells surrounding the necrotic areas. The lungs had sections of consolidation, with evidence of hemorrhage in the bronchi. There was evidence of hemorrhage in some of the kidneys, and the venules and capillaries of the brain were filled with coagulated material [46].

Many early efficacy studies of MARV vaccine candidates used strain 13 guinea pigs, an inbred guinea pig strain requiring virus adaptation to cause disease. MARV-Musoke and RAVV were passaged 8 or 2 times, respectively, through strain 13 guinea pigs, then were plaque purified 3 times on Vero E6 cells, generating a guinea pig-adapted MARV-Musoke (gpa-MARV-Mus) and guinea pig-adapted RAVV (gpa-RAVV) [63]. Both adapted viruses caused disease in strain 13 guinea pigs and viremia was detected on 7 dpi, but only gpa-RAVV was uniformly lethal, with gpa-MARV-Mus having a varying lethality. Strain 13 guinea pigs that survived infection were found to generate protective antibodies, as serum transfer from immune guinea pigs completely protected naïve guinea pigs against both gpa-MARV-Mus and gpa-RAVV [63]. Gpa-MARV-Mus was used in other vaccine studies using strain 13 guinea pigs with promising results, including an Alphavirus replicon vaccine that went on to give complete protection against MARV-Musoke in cynomolgus macaques, though still with varying lethality with gpa-MARV-Mus in strain 13 guinea pigs [64, 65]. In addition, an attenuated gpa-MARV-Mus collected at only 6 passages was shown to be nonlethal and protective in strain 13 guinea pigs but was lethal to Hartley guinea pigs when inoculated at a 10 times lower dose [66]. A similar study inoculated strain 13 guinea pigs with a guinea pig adapted MARV-Ci67 (gpa-MARV-Ci67), gpa-MARV-Mus, and gpa-RAVV, resulting in uniform lethality for gpa-MARV-Ci67 and gpa-RAVV, but non-uniform lethality for gpa-MARV-Mus^)^ [67]. There was advanced necrosis in the liver and lymphocytolysis in the spleen for all three strains tested, with no sign of a maculopapular rash [67]. Strain 13 guinea pigs, due to their inbred nature, are thought to have a less robust immune system, and so outbred Hartley guinea pig models became a more suitable model.

To develop and characterize a MVD model in Hartley guinea pigs, MARV-Angola was serially passaged 4 times in Hartley guinea pigs to develop a guinea pig-adapted MARV-Angola (gpa-MARV-Ang) [68]. Gpa-MARV-Ang caused uniform lethality in Hartley guinea pigs when challenged with 1000 PFU i.p., with death occurring 6–9 dpi. Gpa-MARV-Ang was then used to inoculate Hartley guinea pigs at 5000 PFU, i.p. Viral titers were detected in the liver, kidney, lung, and spleen. The guinea pigs exhibited a similar gross pathology to the previously described Hartley guinea pig study by Simpson et al [46] Just as with the strain 13 guinea pigs, there was no sign of a maculopapular rash. Lymphopenia, thrombocytopenia, basophilia, and eosinophilia were detected at 5 dpi, and continued until endpoint [68]. Beginning on day 7, there was a 10-fold increase of AST and ALT in the serum, and the levels of bilirubin were tripled on the final timepoint. This indication of liver involvement is supported by the presence of MARV antigen detected in Kupffer cells and adjacent hepatocytes starting on 3 dpi, followed by necrotic lesions and leukocytosis in the liver. The spleen showed dendritiform cells positive for MARV antigen scattered throughout the red and white pulp on 3 dpi, with progressive lymphocytosis, hemorrhage, and fibrin accumulation in the white pulp until death [68]. Lymphoid depletion in several lymph nodes and the gastrointestinal tract was observed starting on 5 dpi. In the lungs, MARV antigen positive alveolar macrophages and mononuclear cell were detected at final timepoints, indicating interstitial pneumonia. Blood coagulation times and fibrinogen levels increased throughout infection as protein C activity and tissue factor levels decreased, indicating DIC [68]. There was a significant upregulation of both pro- and anti- inflammatory cytokines starting at 3 dpi. Gpa-MARV-Ang differs from wt MARV-Angola by a single amino acid change in VP40, 2 in VP24, 3 nucleotide changes in non-coding regions, and 2 silent mutations in the polymerase gene L. A similar but independent study by Cross et al. passaged MARV-Angola through guinea pigs to produce a gpa-MARV-Ang, resulting in an adapted virus with a high degree of sequence similarity to the gpa-MARV-Ang previously described [69]. After 9 passages through guinea pigs, all coding and non-coding mutations were the same as Cross et al., except for 1 additional VP24 mutation, and a single additional non-coding region mutation in Cross et al.’s adapted virus that gpa-MARV-Ang lacked [69]. This indicates that the majority of changes observed in both adapted viruses are likely necessary to cause lethal MVD in guinea pigs [70].

### MARV Syrian Golden hamster model

Syrian golden hamsters, much like mice, show no signs of disease when infected with wild-type MARV. STAT2 knock out (STAT2^−/−^) hamsters have a deficient IFN pathway, making them susceptible to lethal MARV infection with multiple strains [71]. STAT2^−/−^ hamsters showed uniform lethality when infected with 10,000 PFU of MARV-Musoke via the i.p. route, showing symptoms of disease starting at 6 dpi. Observed symptoms included lethargy, irregular breathing, weight loss, nasal discharge, abnormal gait, hyperreflexia, and head tilt. There was no evidence of maculopapular rash or significant changes in platelet counts. Lymphocytes decreased initially in disease, followed by a rise in neutrophils, but neither measurement was statistically significant [71]. No blood tests for markers of DIC were performed. MARV was found at high titers in the blood, kidneys, spleen, liver, lymph nodes, and heart, with a moderate titer in the brain. A wide array of cytokines released indicated a strong and dysregulated immune response. Necropsies showed the liver and spleen had necrotic lesions and infiltration of macrophages and neutrophils throughout the red and white pulp of the spleen, with MARV antigen detected throughout both organs. There was diffuse lymphocytolysis in the spleen [71].

Suckling golden hamsters are susceptible to infection with wt MARV, but with nonconclusive and varying results in an analysis of gross pathology of the liver, spleen, and kidneys. The brain, however, showed neuropathology, with swelling, enlargement of blood vessels, and hemorrhage consistently observed. After 9 passages in guinea pigs, 3 in monkeys, and 9 more passages in suckling hamsters, adult golden hamsters showed more typical MARV pathology in the liver, spleen, and kidneys, with non-uniform lethality. The brains of a few adult hamsters showed hemorrhages in the brain and encephalitic lesions. This is the only small animal model to show extensive encephalitis via an inoculation route other than intracerebral [72, 73].

For a different approach, MARV-Angola was passaged three times through Hartley guinea pigs, then five times in hamsters, producing a hamster adapted MARV-Angola (ha-MARV-Ang) [74]. Syrian golden hamsters were inoculated with 1 PFU of ha-MARV-Ang via the i.p. route, resulting in significant weight loss by 5 dpi. The hamsters continued to lose weight and experienced a brief spike in temperature, followed by a drop in temperature just before the death/euthanasia at 8 dpi. A maculopapular rash formed on the bodies, arms, and faces by 7 dpi, along with hemorrhagic symptoms at varying levels; in the small intestine, gastro-duodenal junction, adrenal glands, kidneys, cervical lymph nodes, footpads, joints, and under the skin. Viral titers were detected in most organs, including the liver, spleen, lymph nodes, and brain. Hematological analysis showed leukopenia and lymphopenia in the early stages of disease, with leukocytosis and lymphocytosis in the latest stages of disease [74]. Platelet counts non-significantly decreased initially in infection but spiked on 7–8 dpi. Blood coagulation times (PT, aPTT, and thrombin) increased late in infection, indicating DIC. An analysis of the blood, liver, and spleen showed an increase in both pro- and anti- inflammatory cytokines, indicating a dysregulation of the immune system. Upon necropsy, the livers were pale and swollen, with necrotic lesions and infiltration of neutrophils. MARV antigen was detected in endothelial cells, hepatocytes, and Kupffer cells [74]. Hamster spleens were enlarged and pale, and the red pulp had fibrin deposited throughout and was infiltrated by neutrophils and macrophages. In the white pulp there was widespread necrosis, lymphopenia, and tingible body macrophages. Viral antigen was detected in mononuclear cells early in infection, and extensively in the red pulp late in disease progression. Compared to the wild-type virus, ha-MARV-Ang had 2 amino acid changes in VP40, 1 change in VP35, and 58 silent mutations, most of which occurred in the non-coding region of VP35 [74].

While the hamster is an animal species easier to handle in the BLS4, it lacks a repertoire of reagents for thorough analyses of disease progression and immune responses. Guinea pig reagents are also sparse compared to mice, however, this far the Guinea pig and NHP models are still the most used laboratory animals for this research.

### MARV ferret model

The ferret model has been established as a model for ebolavirus infection [75], however, infection of ferrets with either MARV or RAVV by multiple routes and doses did not lead to development of signs of disease [75, 76]. It appears that the virus will need to be adapted to this species in order to cause disease (Fig. 2).

## Conclusions

MARV infection in humans is often characterized by a swift onset, a high chance of transmission to ill-equipped primary caregivers, and a high mortality rate. These traits, combined with the rural, infrequent, and chaotic nature of most outbreaks make human efficacy trials for MARV vaccines and treatments logistically and ethically challenging. The animal efficacy rule put forward by the US Food and Drug Administration (FDA) states that when human efficacy trials are not feasible, efficacy data from one or more animal models that accurately mimic disease and predict response in humans can be used as evidence of effectiveness [77]. In order to develop and test vaccines and therapeutics for MARV, animal models of MVD must be developed and characterized, and reflect human pathogenesis as accurately as possible. Based on a review of the literature and as described above, it’s clear that the NHP model best recapitulates MVD pathogenesis in humans. However, the animal husbandry burden, financial cost, and ethical concerns surrounding NHPs make them a poor choice for pilot experiments involving untested vaccines and treatments. The easier and more cost-effective approach is to test potential vaccines and therapeutics in small animal models of MVD, to establish some degree of efficacy before moving to NHP studies. The VSV-EBOV vaccine, the first EBOV vaccine with clinical data showing effectiveness in humans [78], was shown to protect mice, guinea pigs, hamsters, and NHPs from EBOV challenge [79–81].

The Syrian golden hamster model of MVD presents an outcome very similar to MVD in humans, but requires an adapted virus, and has a limited array of commercially available reagents and assays. Guinea pigs have more commercially available products, but do not recapitulate some of the important aspects of human MVD (Tables 1 and 2). Mice are incredibly easy to acquire and handle, and have by far the most reagents available, as well as a multitude of transgenic and knockout models. However, mice also present pathology that is furthest from human MVD and are either immunodeficient or require an adapted virus to cause disease. The animal models for MVD reviewed in this paper are effective at recapitulating human MVD pathogenesis in different capacities, and each has its place in the long process of testing vaccines and treatments for licensure. However, there is room for further characterization and development of new animal models of MVD, in order to close the gap between using simple animal models and accuracy of disease progression.

## Data Availability

n/a

## Abbreviations

MARV: Marburg virus
MVD: Marburg virus disease
NHPs: Nonhuman primates
RAVV: Virion protein (VP); Ravn virus
EBOV: Ebola virus
ALT: Alanine aminotransferase
AST: Aspartate aminotransferase
DIC: Disseminated intravascular coagulation
mpi: months post infection
BUN: Blood urea nitrogen
SCID: Severe combined immunodeficient
ma-RAVV: mouse-adapted RAVV
wt: wild-type
i.p.: intraperitoneal
ALP: Alkaline phosphatase
ma-MARV-Ang: mouse adapted MARV-Angola
VSV: Vesicular stomatitis virus
rVSV: recombinant VSV
BLT: Bone marrow, liver, thymus
TKO-BLT: Triple knockout BLT
i.m.: intramuscular
RBCs: Red blood cells
gpa-MARV-Mus: guinea pig adapted MARV-Musoke
gpa-RAVV: guinea pig adapted RAVV
gpa-MARV-Ci67: guinea pig adapted MARV-Ci67
gpa-MARV-Ang: guinea pig adapted MARV-Angola
KO: Knock out
ha-MARV-Ang: hamster adapted MARV-Angola
FDA: Food and Drug Administration
BSL4: Biosafety level 4

## References

[1] Languon S, Quaye O (2019). Filovirus Disease Outbreaks: A Chronological Overview. Virology (Auckl).

[2] Kuhn JH, Adachi T, Adhikari NKJ, Arribas JR, Bah IE, Bausch DG (2019). New filovirus disease classification and nomenclature. Nat Rev Microbiol.

[3] Shi M, Lin XD, Chen X, Tian JH, Chen LJ, Li K (2018). The evolutionary history of vertebrate RNA viruses. Nature.

[4] Feldmann H, Muhlberger E, Randolf A, Will C, Kiley MP, Sanchez A (1992). Marburg virus, a filovirus: messenger RNAs, gene order, and regulatory elements of the replication cycle. Virus Res.

[5] Geisbert TW, Jahrling PB (1995). Differentiation of filoviruses by electron microscopy. Virus Res.

[6] Kuhn JH, Bao Y, Bavari S, Becker S, Bradfute S, Brister JR (2013). Virus nomenclature below the species level: a standardized nomenclature for natural variants of viruses assigned to the family Filoviridae. Arch Virol.

[7] Siegert R, Shu HL, Slenczka W, Peters D, Muller G (1967). On the etiology of an unknown human infection originating from monkeys. Dtsch Med Wochenschr.

[8] Bausch DG, Nichol ST, Muyembe-Tamfum JJ, Borchert M, Rollin PE, Sleurs H (2006). Marburg hemorrhagic fever associated with multiple genetic lineages of virus. N Engl J Med.

[9] Towner JS, Khristova ML, Sealy TK, Vincent MJ, Erickson BR, Bawiec DA (2006). Marburgvirus genomics and association with a large hemorrhagic fever outbreak in Angola. J Virol.

[10] Pigott DM, Golding N, Mylne A, Huang Z, Weiss DJ, Brady OJ (2015). Mapping the zoonotic niche of Marburg virus disease in Africa. Trans R Soc Trop Med Hyg.

[11] Schuh AJ, Amman BR, Towner JS (2017). Filoviruses and bats. Microbiol Aust.

[12] Bertherat E, Talarmin A, Zeller H (1999). Democratic Republic of the Congo: between civil war and the Marburg virus. Internateional Committee of Technical and Scientific Coordination of the Durba epidemic. Med Trop (Mars).

[13] Centers for Disease C, Prevention (2009). Imported case of Marburg hemorrhagic fever - Colorado, 2008. MMWR Morb Mortal Wkly Rep.

[14] Timen A, Koopmans MP, Vossen AC, van Doornum GJ, Gunther S, van den Berkmortel F (2009). Response to imported case of Marburg hemorrhagic fever, the Netherland. Emerg Infect Dis.

[15] Towner JS, Pourrut X, Albarino CG, Nkogue CN, Bird BH, Grard G (2007). Marburg virus infection detected in a common African bat. PLoS One.

[16] Amman BR, Jones ME, Sealy TK, Uebelhoer LS, Schuh AJ, Bird BH (2015). Oral shedding of Marburg virus in experimentally infected Egyptian fruit bats (Rousettus aegyptiacus). J Wildl Dis.

[17] Paweska JT, Jansen van Vuren P, Fenton KA, Graves K, Grobbelaar AA, Moolla N (2015). Lack of Marburg Virus Transmission From Experimentally Infected to Susceptible In-Contact Egyptian Fruit Bats. J Infect Dis.

[18] Paweska Janusz T., Jansen van Vuren Petrus, Masumu Justin, Leman Patricia A., Grobbelaar Antoinette A., Birkhead Monica, Clift Sarah, Swanepoel Robert, Kemp Alan (2012). Virological and Serological Findings in Rousettus aegyptiacus Experimentally Inoculated with Vero Cells-Adapted Hogan Strain of Marburg Virus. PLoS ONE.

[19] Jones Megan, Amman Brian, Sealy Tara, Uebelhoer Luke, Schuh Amy, Flietstra Timothy, Bird Brian, Coleman-McCray JoAnn, Zaki Sherif, Nichol Stuart, Towner Jonathan (2019). Clinical, Histopathologic, and Immunohistochemical Characterization of Experimental Marburg Virus Infection in A Natural Reservoir Host, the Egyptian Rousette Bat (Rousettus aegyptiacus). Viruses.

[20] Martini GA (1973). Marburg virus disease. Postgrad Med J.

[21] Kissling RE, Murphy FA, Henderson BE (1970). Marburg virus. Ann N Y Acad Sci.

[22] Glaze ER, Roy MJ, Dalrymple LW, Lanning LL (2015). A comparison of the pathogenesis of Marburg virus disease in humans and nonhuman Primates and evaluation of the suitability of these animal models for predicting clinical efficacy under the 'Animal Rule'. Comp Med.

[23] Gear JS, Cassel GA, Gear AJ, Trappler B, Clausen L, Meyers AM (1975). Outbreak of Marburg virus disease in Johannesburg. Br Med J.

[24] Smith DH, Johnson BK, Isaacson M, Swanapoel R, Johnson KM, Killey M (1982). Marburg-virus disease in Kenya. Lancet.

[25] Martines RB, Ng DL, Greer PW, Rollin PE, Zaki SR (2015). Tissue and cellular tropism, pathology and pathogenesis of Ebola and Marburg viruses. J Pathol.

[26] Roberts A, Kemp C (2001). Ebola and Marburg hemorrhagic fevers. J Am Acad Nurse Pract.

[27] Martini GA (1969). Marburg agent disease: in man. Trans R Soc Trop Med Hyg.

[28] Bechtelsheimer H, Korb G, Gedigk P (1972). The morphology and pathogenesis of "Marburg virus" hepatitis. Hum Pathol.

[29] Stille W, Böhle E, Martini GA, Siegert R (1971). Clinical Course and Prognosis of Marburg Virus (“Green-Monkey”) Disease. Marburg Virus Disease.

[30] Havemann K, Schmidt HA, Martini GA, Siegert R (1971). Haematological Findings in Marburg Virus Disease: Evidence for Involvement of the Immunological System. Marburg Virus Disease.

[31] Stonier SW, Herbert AS, Kuehne AI, Sobarzo A, Habibulin P, Dahan CVA (2017). Marburg virus survivor immune responses are Th1 skewed with limited neutralizing antibody responses. J Exp Med.

[32] Geisbert TW, Hensley LE, Gibb TR, Steele KE, Jaax NK, Jahrling PB (2000). Apoptosis induced in vitro and in vivo during infection by Ebola and Marburg viruses. Lab Investig.

[33] Geisbert TW, Jaax NK (1998). Marburg hemorrhagic fever: report of a case studied by immunohistochemistry and electron microscopy. Ultrastruct Pathol.

[34] Geisbert TW, Strong JE, Feldmann H (2015). Considerations in the use of nonhuman primate models of Ebola virus and Marburg virus infection. J Infect Dis.

[35] Geisbert TW, Daddario-DiCaprio KM, Geisbert JB, Young HA, Formenty P, Fritz EA (2007). Marburg virus Angola infection of rhesus macaques: pathogenesis and treatment with recombinant nematode anticoagulant protein c2. J Infect Dis.

[36] Fernando L, Qiu X, Melito PL, Williams KJ, Feldmann F, Feldmann H (2015). Immune response to Marburg virus Angola infection in nonhuman Primates. J Infect Dis.

[37] Alves DA, Glynn AR, Steele KE, Lackemeyer MG, Garza NL, Buck JG (2010). Aerosol exposure to the Angola strain of Marburg virus causes lethal viral hemorrhagic fever in cynomolgus macaques. Vet Pathol.

[38] Johnston SC, Lin KL, Twenhafel NA, Raymond JL, Shamblin JD, Wollen SE (2015). Dose response of MARV/Angola infection in Cynomolgus macaques following IM or aerosol exposure. PLoS One.

[39] MG HR, SR MGA, Experimental Infection of Monkeys with the Marburg Virus (1971). Marburg Virus Disease.

[40] Lin KL, Twenhafel NA, Connor JH, Cashman KA, Shamblin JD, Donnelly GC (2015). Temporal characterization of Marburg virus Angola infection following aerosol challenge in rhesus macaques. J Virol.

[41] Woolsey C, Geisbert JB, Matassov D, Agans KN, Borisevich V, Cross RW (2018). Postexposure Efficacy of Recombinant Vesicular Stomatitis Virus Vectors Against High and Low Doses of Marburg Virus Variant Angola in Nonhuman Primates. J Infect Dis.

[42] Mire CE, Geisbert JB, Borisevich V, Fenton KA, Agans KN, Flyak AI (2017). Therapeutic treatment of Marburg and Ravn virus infection in nonhuman primates with a human monoclonal antibody. Sci Transl Med.

[43] Dye JM, Warfield KL, Wells JB, Unfer RC, Shulenin S, Vu H (2016). Virus-like particle vaccination protects nonhuman Primates from lethal aerosol exposure with Marburgvirus (VLP vaccination protects macaques against aerosol challenges). Viruses.

[44] Hensley LE, Alves DA, Geisbert JB, Fritz EA, Reed C, Larsen T (2011). Pathogenesis of Marburg hemorrhagic fever in cynomolgus macaques. J Infect Dis.

[45] Geisbert TW, Geisbert JB, Leung A, Daddario-DiCaprio KM, Hensley LE, Grolla A (2009). Single-injection vaccine protects nonhuman primates against infection with Marburg virus and three species of ebola virus. J Virol.

[46] Simpson DI, Zlotnik I, Rutter DA (1968). Vervet monkey disease. Experiment infection of Guinea pigs and monkeys with the causative agent. Br J Exp Pathol.

[47] Simpson DI (1969). Marburg agent disease: in monkeys. Trans R Soc Trop Med Hyg.

[48] Marzi A, Menicucci AR, Engelmann F, Callison J, Horne EJ, Feldmann F (2018). Protection against Marburg virus using a recombinant VSV-vaccine depends on T and B cell activation. Front Immunol.

[49] Furuyama W, Marzi A (2019). Ebola virus: pathogenesis and countermeasure development. Annu Rev Virol.

[50] Carrion R, Ro Y, Hoosien K, Ticer A, Brasky K, de la Garza M (2011). A small nonhuman primate model for filovirus-induced disease. Virology.

[51] Smither SJ, Nelson M, Eastaugh L, Laws TR, Taylor C, Smith SA (2013). Experimental respiratory Marburg virus haemorrhagic fever infection in the common marmoset (Callithrix jacchus). Int J Exp Pathol.

[52] Bray M (2001). The role of the type I interferon response in the resistance of mice to filovirus infection. J Gen Virol.

[53] Raymond J, Bradfute S, Bray M (2011). Filovirus infection of STAT-1 knockout mice. J Infect Dis.

[54] Warfield KL, Alves DA, Bradfute SB, Reed DK, VanTongeren S, Kalina WV (2007). Development of a model for marburgvirus based on severe-combined immunodeficiency mice. Virol J.

[55] Warfield KL, Bradfute SB, Wells J, Lofts L, Cooper MT, Alves DA (2009). Development and characterization of a mouse model for Marburg hemorrhagic fever. J Virol.

[56] Qiu X, Wong G, Audet J, Cutts T, Niu Y, Booth S (2014). Establishment and characterization of a lethal mouse model for the Angola strain of Marburg virus. J Virol.

[57] Wei H, Audet J, Wong G, He S, Huang X, Cutts T (2017). Deep-sequencing of Marburg virus genome during sequential mouse passaging and cell-culture adaptation reveals extensive changes over time. Sci Rep.

[58] Valmas C, Basler CF (2011). Marburg virus VP40 antagonizes interferon signaling in a species-specific manner. J Virol.

[59] Feagins AR, Basler CF (2015). Amino acid residue at position 79 of Marburg virus VP40 confers interferon antagonism in mouse cells. J Infect Dis.

[60] Mestas J, Hughes CC (2004). Of mice and not men: differences between mouse and human immunology. J Immunol.

[61] Marzi A, Kercher L, Marceau J, York A, Callsion J, Gardner DJ (2015). Stat1-deficient mice are not an appropriate model for efficacy testing of recombinant vesicular stomatitis virus-based Filovirus vaccines. J Infect Dis.

[62] Lavender KJ, Williamson BN, Saturday G, Martellaro C, Griffin A, Hasenkrug KJ (2018). Pathogenicity of Ebola and Marburg Viruses Is Associated With Differential Activation of the Myeloid Compartment in Humanized Triple Knockout-Bone Marrow, Liver, and Thymus Mice. J Infect Dis.

[63] Hevey M, Negley D, Geisbert J, Jahrling P, Schmaljohn A (1997). Antigenicity and vaccine potential of Marburg virus glycoprotein expressed by baculovirus recombinants. Virology.

[64] Hevey M, Negley D, Pushko P, Smith J, Schmaljohn A (1998). Marburg virus vaccines based upon alphavirus replicons protect Guinea pigs and nonhuman primates. Virology.

[65] Warfield KL, Swenson DL, Negley DL, Schmaljohn AL, Aman MJ, Bavari S (2004). Marburg virus-like particles protect Guinea pigs from lethal Marburg virus infection. Vaccine.

[66] Hevey M, Negley D, VanderZanden L, Tammariello RF, Geisbert J, Schmaljohn C (2001). Marburg virus vaccines: comparing classical and new approaches. Vaccine.

[67] Swenson DL, Warfield KL, Larsen T, Alves DA, Coberley SS, Bavari S (2008). Monovalent virus-like particle vaccine protects Guinea pigs and nonhuman primates against infection with multiple Marburg viruses. Expert Rev Vaccines.

[68] Wong G, Cao WG, He SH, Zhang ZR, Zhu WJ, Moffat E (2018). Development and characterization of a Guinea pig model for Marburg virus. Zool Res.

[69] Cross RW, Fenton KA, Geisbert JB, Ebihara H, Mire CE, Geisbert TW (2015). Comparison of the pathogenesis of the Angola and Ravn strains of Marburg virus in the outbred Guinea pig model. J Infect Dis.

[70] Lofts LL, Ibrahim MS, Negley DL, Hevey MC, Schmaljohn AL (2007). Genomic differences between Guinea pig lethal and nonlethal Marburg virus variants. J Infect Dis.

[71] Atkins C, Miao J, Kalveram B, Juelich T, Smith JK, Perez D (2018). Natural History and Pathogenesis of Wild-Type Marburg Virus Infection in STAT2 Knockout Hamsters. J Infect Dis.

[72] Zlotnik I, Simpson DI (1969). The pathology of experimental vervet monkey disease in hamsters. Br J Exp Pathol.

[73] Zlotnik I (1969). Marburg agent disease: pathology. Trans R Soc Trop Med Hyg.

[74] Marzi A, Banadyga L, Haddock E, Thomas T, Shen K, Horne EJ (2016). A hamster model for Marburg virus infection accurately recapitulates Marburg hemorrhagic fever. Sci Rep.

[75] Cross RW, Mire CE, Agans KN, Borisevich V, Fenton KA, Geisbert TW (2018). Marburg and Ravn Viruses Fail to Cause Disease in the Domestic Ferret (*Mustela putorius furo*). J Infect Dis.

[76] Wong G, Zhang Z, He S, de La Vega MA, Tierney K, Soule G (2018). Marburg and Ravn Virus Infections Do Not Cause Observable Disease in Ferrets. J Infect Dis.

[77] Crawford LM, Food and Drug Administration H (2002). New drug and biological drug products; evidence needed to demonstrate effectiveness of new drugs when human efficacy studies are not ethical or feasible. Federal Register.

[78] Henao-Restrepo AM, Longini IM, Egger M, Dean NE, Edmunds WJ, Camacho A (2015). Efficacy and effectiveness of an rVSV-vectored vaccine expressing Ebola surface glycoprotein: interim results from the Guinea ring vaccination cluster-randomised trial. Lancet.

[79] Wong G, Audet J, Fernando L, Fausther-Bovendo H, Alimonti JB, Kobinger GP (2014). Immunization with vesicular stomatitis virus vaccine expressing the Ebola glycoprotein provides sustained long-term protection in rodents. Vaccine.

[80] Tsuda Y, Safronetz D, Brown K, LaCasse R, Marzi A, Ebihara H (2011). Protective efficacy of a bivalent recombinant vesicular stomatitis virus vaccine in the Syrian hamster model of lethal Ebola virus infection. J Infect Dis.

[81] Jones SM, Feldmann H, Stroher U, Geisbert JB, Fernando L, Grolla A (2005). Live attenuated recombinant vaccine protects nonhuman primates against Ebola and Marburg viruses. Nat Med.

